# Hair Dyes in Pregnancy: A Systematic Review and Meta‐Analysis

**DOI:** 10.1155/jp/9361797

**Published:** 2026-09-01

**Authors:** Stefano Uccella, Chiara Casprini, Giada Scaggiante, Nikolaos Papadopoulos, Pier Carlo Zorzato, Giulia Biancotto, Mariachiara Bosco, Simone Garzon

**Affiliations:** ^1^ Unit of Obstetrics and Gynecology, Department of Surgery, Dentistry, Pediatrics, and Gynecology, AOUI Verona, University of Verona, Verona, Italy, univr.it

**Keywords:** carcinogen exposure, chemical exposure, cosmetic, fetal malformation, occupational hazard, teratogens, toxin

## Abstract

**Objective:**

The main objective is to investigate the association between periconceptional or pregnancy exposure to hair dyes and obstetric and pediatric outcomes.

**Methods:**

A systematic literature search was conducted in Scopus, PubMed/MEDLINE, Web of Science, and CINAHL Ultimate from database inception to August 31, 2024. Studies were included based on PECO criteria: pregnant women and their newborns (population), exposure to hair dyes during periconception or pregnancy (exposure), no exposure (comparator), and any obstetric or pediatric complication (outcome). After screening 311 records and removing duplicates, 179 abstracts were reviewed, 29 full texts assessed, and 20 observational studies included. Four reviewers assessed bias and extracted data. Meta‐analyses were performed using a random‐effects model when appropriate.

**Results:**

Few studies distinguished hair dye types or exposure timing. Periconceptional or pregnancy hair dye exposure was reported to be associated with stillbirth, low birth weight (Pooled OR 2.30; 95% CI 1.24–4.25), and low hormone levels; in the offspring, with allergic rhinitis, leukemia, neuroblastoma (pooled OR 1.79; 95% CI 1.20–2.68), Wilms tumor (pooled OR 6.00; 95% CI 1.22–29.46), germ cell tumors, and membranous ventricular septal defect. No association was observed with asthma (pooled OR 1.19; 95% CI 0.86–1.65), childhood brain tumors (pooled OR 1.06; 95% CI 0.88–1.28), or mental retardation. Based on the GRADE assessment, the certainty of evidence was low or very low.

**Conclusions:**

Current observational evidence suggests a potential association between periconceptional or pregnancy exposure to hair dyes and some adverse obstetric and pediatric outcomes. However, given the low and very low certainty of evidence and the retrospective nature of most included studies, these findings are hypothesis‐generating and preclude causal inference. Further prospective studies are needed to confirm these preliminary associations and define the specific chemicals involved.

## 1. Introduction

Natural hair color depends on the type and amount of melanin content within the hair cortex, and gray hairs are a typical manifestation of aging [1]. The constant increase in women′s age at the first pregnancy raises the proportion of women exposed to hair dyes in the periconceptional period or who question their use during pregnancy [2]. It has been estimated that more than one‐third of women over 18 use some type of hair dye in Europe and the United States [3].

Online information, including that provided by public health agencies, generally conveys a reassuring perspective. For instance, an NHS information sheet addressing the question “Is it safe to use hair dye when I′m pregnant or breastfeeding?” concludes that hair dyes are considered safe, albeit acknowledging the limited scope of available research. Although exposure to high doses of certain chemicals is noted to be potentially harmful, the concentration of such chemicals in hair dyes is emphasized as being minimal. The information sheet further advises reducing risks by wearing gloves, limiting the application time, and ensuring proper ventilation during use [4]. However, a meta‐analysis examining reproductive disorders among hairdressers identified an association with adverse reproductive and obstetric outcomes [5].

Hair dyes are heterogeneous and can be classified based on their origin (vegetable, mineral, metallic, or synthetic) and length of effect (Table 1) [1]. Permanent dyes are mainly synthetic, last indefinitely, and act through oxidation, among which aromatic amines, such as p‐phenylenediamine (PPD), represent the main class [7]. Previous studies have already demonstrated the toxicological properties of PPD [8, 9], such as the induction of apoptosis by increasing reactive oxygen species [6]. Additionally, PPD can penetrate the skin or be absorbed by inhalation [10]. Unlike PPD, semipermanent dyes contain very low‐weight molecules that penetrate the cortex without oxidative processes; [10] temporary dyes consist of large molecules that deposit superficially and are removed by a few washings, making them less likely to be absorbed [7]. However, some of these dyes, such as those of mineral or metallic origin, are recognized as potentially toxic.

**Table 1:** Hair dyes classification by origin and length of effect.

Origin	Length of effect	Notes
Vegetal	Fade quickly	Nontoxic (e.g., henna, chamomile, and quinine)

Mineral or metallic	Weeks/months	Can be toxic

Synthetic	Temporary dyes (single wash)	High molecular weight molecules that deposit on the cuticle
	Semipermanent dyes (6–12 washings)	Low molecular weight molecules and is introduced superficially into the cortex
	Permanent dyes (indefinite length)	Very low molecular weight molecules, it is introduced deep into the cortex
	Decolorant dyes	

*Note: Source:* Modified from Guerra‐Tapia A, Gonzalez‐Guerra E. Hair Cosmetics: Dyes. Actas Dermo‐Sifiliográficas (English Edition). Two thousand and fourteen Nov; 105 [6]:833–9.

On this basis, this systematic review and meta‐analysis was specifically conducted to summarize the available evidence on the association between periconceptional and pregnancy exposure to hair dyes and adverse obstetric and pediatric outcomes.

## 2. Materials and Methods

### 2.1. Eligibility Criteria

Before starting the online search, this systematic review was planned considering the population of interest, exposure and comparator definitions, outcome measures, and study eligibility criteria (PROSPERO CRD42024580586). The review and meta‐analysis were conducted following the Cochrane Handbook for Systematic Reviews of Interventions and reported based on the Preferred Reporting Items for Systematic Reviews and Meta‐Analyses (PRISMA) [11, 12] and the Meta‐analysis of Observational Studies in Epidemiology (MOOSE) guidelines [13].

### 2.2. Information Sources

A systematic literature search was conducted in Scopus, PubMed/MEDLINE, Web of Science, and CINAHL Ultimate from database inception to August 31, 2024, by an expert librarian (Biblioteca Meneghetti, University of Verona)

### 2.3. Search Strategy

The search strategy included the combinations of the medical terms: hair dye, pregnancy, and conception (File S1). The references of all included records were systematically reviewed.

### 2.4. Study Selection

We included all references reporting studies selected based on prespecified PECO (population, exposure, comparator, and outcome) criteria [14, 15] and aimed to explore the association between hair dye exposure during periconceptional and pregnancy periods and obstetrics or pediatric outcomes: population, pregnant women and their newborns; exposure, contact with any hair dye during the periconceptional period or pregnancy; comparator, absent contact with any hair dye during the periconceptional period or pregnancy; and outcomes, any obstetric complication or pediatric pathology. Outcomes eligibility required reporting at least one of the outcomes of interest. No selection criteria were applied to the study design. We included studies presented in any publication format (e.g., full reports or conference abstracts). We excluded review articles, records not presenting study data, and animal studies. No language exclusion criterion was applied; translation with Google Translator was planned for non‐English titles, abstracts, or full‐text records [16].

### 2.5. Data Extraction

Two reviewers independently screened titles and abstracts of all identified records. Full texts of selected records were independently assessed for eligibility by other two reviewers. Any disagreement was solved with a senior investigator. Four reviewers blindly extracted data using a standardized form: first author, publication year, research setting, study design, population, type of hair dye, details of exposure, outcomes measures with details on assessment and definition, and items for quality evaluation. We gathered details regarding the hair dye composition, origin (vegetable, mineral, metallic, or synthetic), and length of effect (permanent, semipermanent, and temporary). Exposure information included the timing (periconceptional, first, second, and third trimester), reason for exposure (work vs. personal use), and frequency. Outcomes comprise any deviation from physiologic pregnancy and healthy offspring.

### 2.6. Assessment of Risk of Bias and Evidence Certainty

Four reviewers independently assessed the risk of bias in included studies according to the Newcastle–Ottawa Scale. Any disagreement was resolved by reexamining the article with a further reviewer. The GRADE framework was used to assess the certainty of the evidence. Observational studies started at low certainty according to GRADE guidance for prognostic/exposure evidence. Certainty was downgraded based on risk of bias, inconsistency, indirectness, and imprecision, and was upgraded when a dose–response gradient was identified [17].

### 2.7. Data Synthesis

A meta‐analysis was planned in case two or more studies were identified investigating the same exposure (type of hair dye and timing of exposure) and outcomes in a comparable population. The statistical analyses were prespecified, including subgroup analyses. Formal sensitivity analyses were not feasible because only a small number of studies were available for most pooled outcomes. Meta‐analyses of odds ratios (ORs) or risk ratios (RRs) were planned to use the random‐effects model, given that the assumption of having a common treatment effect for all included studies required by the fixed‐effect model was not expected. Heterogeneity was planned to be quantified using the *I* ^2^ tests; *I* ^2^ less than 25% was considered low, and *I* ^2^ more than 75% was considered high. Publication bias was assessed by the Funnel plot inspection when at least 10 studies were identified. All analyses were two‐tailed with a statistical significance threshold of *p* = 0.05. Meta‐analyses were performed using Review Manager (RevMan) Version 5.4 (The Cochrane Collaboration, 2020).

## 3. Results

### 3.1. Study Selection, Characteristics, and Risk of Bias Assessment

**Figure 1:**
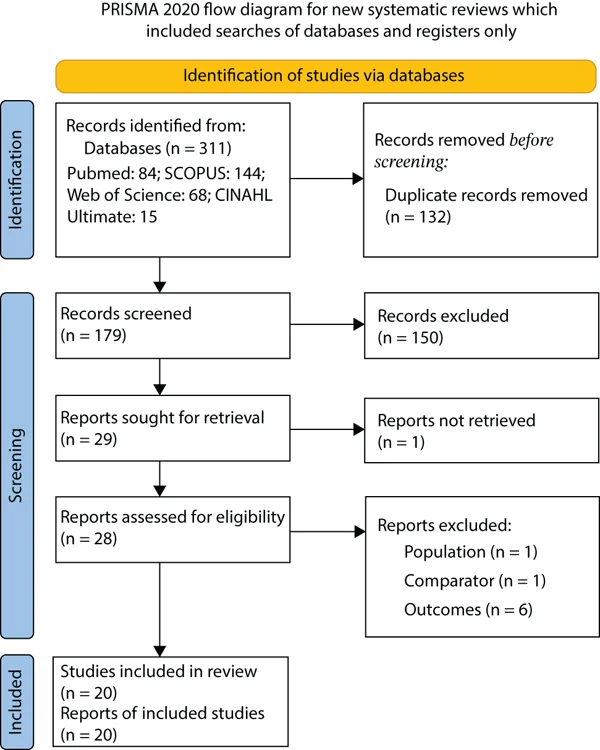
PRISMA flowchart of study selection.

The initial literature search identified 311 records. After duplicate removal and titles/abstract screening, 29 records underwent full‐text evaluation. Six documents were excluded because they were missing the outcomes of interest [18–23], one because it included only women exposed during breastfeeding [24], one was without a comparator [25], and one was excluded due to full‐text unavailability with failure to contact authors [26]. Finally, 20 records met the inclusion criteria corresponding to 20 studies (Figure 1 and Table S1). Two studies were conducted on the same national register but investigated different outcomes [27, 28]. Translation of the full text was required only in one of the studies excluded for missing the outcomes of interest [19].

Most studies were retrospective and had a cohort or case‐control design; only one study provided data from a prospective cohort. All studies assessed the hair dye exposure by conducting a questionnaire survey and defining the exposure as the use of any hair dye. Only a minority focused on a specific type of hair dye or provided results stratified by hair dye categories. Investigated outcomes were heterogeneous and included obstetric complications—such as fetal death, low birth weight (LBW), and other neonatal complications—and pediatric pathologies—such as allergic disease, leukemia, neuroblastoma (NB), Wilms tumor (WT), childhood brain tumors (CBTs), germinal cell tumors (GCTs), structural congenital heart defect, and mental retardation; one study investigated hormone levels in pregnant women. Two or more studies examined the association between hair dye exposure and the risk of LBW, asthma, leukemia, NB, WT, CBTs, and GCTs in the offspring (Table S1).

### 3.2. Risk of Bias and Evidence Certainty Assessment

The risk of bias assessment for each study performed with the Newcastle–Ottawa Scale is reported in Table S2. The selection domain was considered fully satisfied (four stars) by 10 out of 20 studies; studies that did not achieve four stars failed in the case definition or the selection of controls. The comparability domain was considered satisfied in all studies. All but three studies satisfied all three points of the outcome domain; three studies did not fulfill the blinding for the case‐control status of the interviewer.

### 3.3. Synthesis of Results: Obstetric Outcomes

#### 3.3.1. Fetal Death

The association between fetal death and hair dye exposure was investigated by a retrospective study using nationwide birth data from the Japan Environment and Children′s Study (JECS), a prospective birth cohort recruited between January 2011 and March 2014 [27]. Participants were queried about the frequency of chemical substance use, including hair dye, during the first and second/third trimester of pregnancy. Weekly occupational exposure to hair dye exceedingly once a week from the first to the second/third trimester was associated with fetal death in the adjusted model (adjusted odds ratio [aOR] for 1–6 times a week vs. never was 4.92 [95% confidence interval [95% CI] 1.53–15.78]; OR for every day vs. never was 7.59 [95% CI 2.32–24.85]). The dose–response analysis showed a growing risk with the increase in occupational exposure. Conversely, the personal use of hair dye was not associated with fetal death. The certainty of evidence was low, indicating that hair dye exposure during the first and second/third trimester of pregnancy may increase the risk of fetal death compared with no exposure (Table S3).

#### 3.3.2. Low birth weight and Neonatal Complications

A nested case‐control study involved 4978 full‐term infant‐mother pairs and revealed an association between pre‐pregnancy hair dye exposure and LBW (aOR 1.71; 95% CI 1.01–2.92) [29]. OR was higher in women with BMI < 18.5 kg/m^2^ (3.07 vs. 2.53, *p* trend = 0.015) and in those with irregular menstruation (4.53 vs. 2.07, *p* trend = 0.05) than those with only one risk factor.

A retrospective cohort study involving 2040 pregnant women observed a higher rate of LBW among those who dyed hair preconceptionally or in the third trimester than women never exposed or exposed in the first or second trimesters (*p* < 0.001) [30]. The investigation of other neonatal complications did not show statistically significant associations [30].

**Figure 2:**
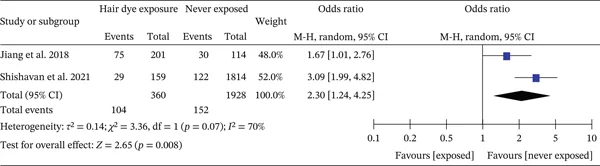
Forest plot of comparison: preconceptional exposure to hair dye versus never exposure; outcome: low birth weight.

Two studies provided a comparison between preconceptional exposure and LBW. Pooled OR for LBW among women preconceptionally exposed to hair dye compared to those never exposed was 2.30 (95% CI 1.24–4.25; *I* ^2^ = 70*%*; chi^2^ *p* value = 0.07) (Figure 2). The certainty of evidence was low, indicating that hair dye exposure during preconception may increase the risk of LBW compared with no exposure (Table S3).

#### 3.3.3. Maternal Hormones

A prospective cohort study examined the association between self‐reported hair dye and other personal care product use and sex‐steroid hormone levels in pregnant women from Puerto Rico [31]. Use of hair dyes, bleach, relaxers, and mousse was associated with lower levels of sex steroid hormones than non‐users: Sex hormone‐binding globulin (SHBG) (%*Δ*: −7.1 [95% CI −12.4 to −1.8]), estriol (E3) (%*Δ*: −23.2 [95% CI −32.2 to −13.0]), progesterone (%*Δ*: −21.5 [95% CI −29.4 to −12.9]), and testosterone (%*Δ*: −21.5 [95% CI −33.1 to –7.8]). The certainty of evidence was very low, indicating that whether hair dye exposure during pregnancy reduces hormone levels compared with no exposure is very uncertain (Table S3).

### 3.4. Synthesis of Results: Pediatric Outcomes

#### 3.4.1. Allergic Diseases

A retrospective cohort study involving 77,303 participants from the JECS investigated the association between hair dye exposure in pregnancy and the occurrence of allergic diseases at 3 years (food allergy, asthma, atopic dermatitis, and allergic rhinitis) [28]. In univariate analyses, home or occupational exposure to hair dyes during pregnancy was significantly associated with asthma (OR 1.15 [95% CI 1.07–1.24] for home and OR 1.18 [95% CI 1.08–1.28] for occupational) and doctor‐diagnosed allergic rhinitis (OR 1.12; 95% CI 1.02–1.22). However, multivariable analysis confirmed only the association between the “quite often” at‐home use and doctor‐diagnosed allergic rhinitis at 3 years of age (aOR for “quite often” vs. “never” 1.78; 95% CI 1.22–2.60).

Partially consistent findings were provided by a retrospective cohort study involving 1199 mother–child pairs in the Kyushu Okinawa Maternal and Child Health Study [32]. Maternal hair dye exposure in pregnancy increased neonatal wheeze risk (aOR 1.44, 95% CI 1.02–2.02) and doctor‐diagnosed asthma at 5 years (aOR 1.51, 95% CI 1.00–2.25).

Pooling the data, women exposed to hair dye in pregnancy than never exposed had a pooled OR for asthma in the offspring of 1.19 (95% CI 0.86–1.65; *I* ^2^ = 63*%*; chi^2^ *p* value = 0.10) (Figure S1). The certainty of evidence was very low, indicating that whether hair dye exposure during pregnancy affects the risk of asthma in the offspring compared with no exposure is very uncertain (Table S3).

#### 3.4.2. Leukemia

Two case‐control studies explored the association between pregnancy exposure to hair dyes or hair straightening cosmetics and leukemia in the offspring. A multicenter retrospective hospital‐based case‐control study was conducted in Brazil between 1999 and 2007 [33], including 176 children under 2 years old with acute lymphocytic leukemia (ALL), 55 with acute myeloid leukemia (AML), and 419 controls. Maternal exposure to hair dyes or hair straightening cosmetics in the first trimester had an aOR of 1.78 (95% CI 1.13–2.81) for ALL. Maternal exposure during breastfeeding had an aOR of 2.43 (95% CI 1.13–5.22) for AML. A case‐control study conducted in Iran between 2015 and 2018 involved 125 children younger than 15 years with ALL [34]. Cases were matched with 130 controls by age, gender, and residence location. No association between hair dye exposure during pregnancy and ALL was observed (OR 0.87; 95% CI 0.32–2.37).

A meta‐analysis was not performed due to excessive differences in the age of the pediatric population. The certainty of evidence was very low, indicating that whether hair dye exposure during pregnancy affects the risk of leukemia in the offspring compared with no exposure is very uncertain (Table S3).

#### 3.4.3. Neuroblastoma

Three retrospective case‐control studies explored the association between hair dye exposure during pregnancy and NB in the offspring. An Italian population‐based study used the SETIL (Italian epidemiological study on the etiology of childhood leukemia, lymphoma, and NB) database [35]. The study included 1044 healthy controls and 153 cases. Exposure to permanent hair dyes in the first two trimesters was associated with a higher risk of NB (OR 2.0). The association was more robust in the 0–17‐month‐old age group (OR 5.5; 95% CI 1.0–29.3). The American case‐control study included 538 cases and 504 controls [36]. A higher risk of NB was associated with any hair dye exposure during pregnancy (OR 1.6; 95% CI 1.1–2.2) but not before pregnancy (OR 1.4; 95% CI 0.9–2.2). The use of any hair dye in the month before or during pregnancy was associated with a moderately increased risk of NB (aOR 1.6; 95% CI 1.2–2.2), with a higher risk for temporary or semipermanent hair dye use (OR 2.0; 95% CI 1.1–3.7) than permanent dyes (OR 1.4; 95% CI 1.0–2.0). A matched case‐control study of prenatal risk factors for NB was conducted in Philadelphia, including 104 cases and 104 controls [37]. Exposure to hair coloring products during pregnancy was reported significantly more often by cases than controls (OR 3.0; 95% CI 1.64–5.48).

**Figure 3:**
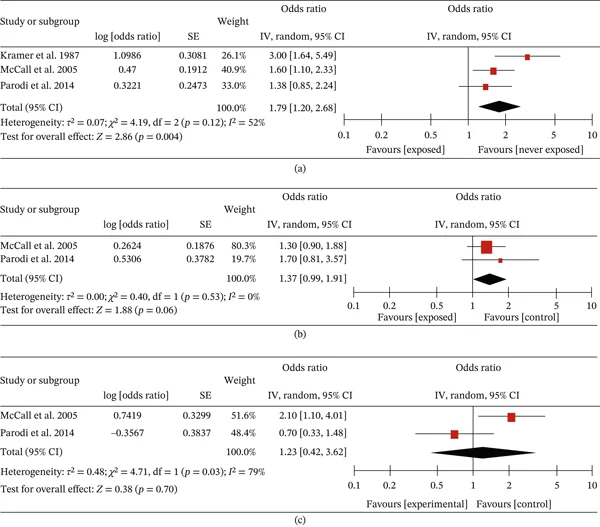
Forest plot of comparison: exposure to hair dye before or during pregnancy versus never exposure; outcome: neuroblastoma. (a) Any hair dye, (b) permanent hair dyes, and (c) temporary hair dyes.

Pooled OR for NB in women exposed to any hair dye before or during pregnancy than never exposed was 1.79 (95% CI 1.20–2.68; *I* ^2^ = 52*%*; chi^2^ *p* value = 0.12) (Figure 3a). The pooled analysis limited to permanent hair dyes reported a pooled OR for NB in exposed women before or during pregnancy than never exposed of 1.37 (95% CI 0.99–1.91; *I* ^2^ = 0*%*; chi^2^ *p* value = 0.53) (Figure 3b), whereas the pooled OR for temporary dyes was 1.23 (95% CI 0.42–3.62; *I* ^2^ = 79*%*; chi^2^ *p* value = 0.03) (Figure 3c). The certainty of evidence was very low, indicating that whether hair dye exposure before and during pregnancy increases the risk of NB in the offspring compared with no exposure is very uncertain (Table S3).

#### 3.4.4. Wilms tumor

A retrospective case‐control study (88 matched pairs) found maternal use of hair coloring products in the year before childbirth associated with a nearly fourfold increased risk of WT (OR 3.6; 95% CI 1.4–10.2) [38]. This association was stronger if evaluated among cases and controls younger than 2 years (OR 15; *p* = 0.001). A more recent retrospective study (200 cases and 233 matched controls) did not confirm this association [39], although the OR was higher in the subgroup of children younger than 2 years (OR 2.92 [CI 0.91–9.33]).

**Figure 4:**
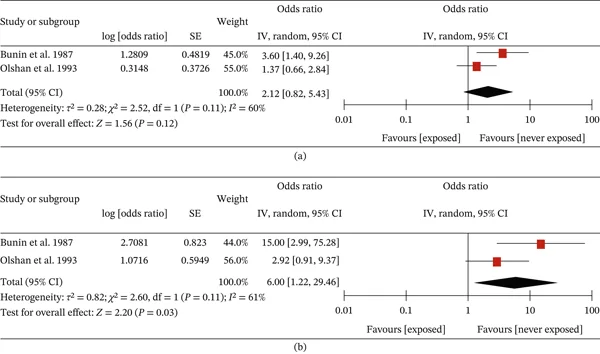
Forest plot of comparison: exposure to hair dye before or during pregnancy versus never exposure; outcome: Wilms tumor. (a) Any age at diagnosis and (b) younger than 2 years at diagnosis.

Pooled OR for WT among those exposed to hair dye before or during pregnancy was 2.12 (95% CI 0.82–5.43; *I* ^2^ = 60*%*; chi^2^ *p* value = 0.11) (Figure 4a); limited to cases and controls younger than 2 years, the pooled OR for WT was 6.00 (95% CI 1.22–29.46; *I* ^2^ = 61*%*; chi^2^ *p* value = 0.11) (Figure 4b). The certainty of evidence was very low, indicating that whether hair dye exposure before or during pregnancy increases the risk of WT in the offspring compared with no exposure is very uncertain (Table S3).

#### 3.4.5. Childhood brain tumors

In a case‐control study (321 matched case–control pairs) on CBTs, maternal use of hair dyes during pregnancy was not associated with astrocytic glioma (OR 0.7; 95% CI 0.3–1.6) or Primitive neuroectodermal tumor of the brain (PNET) (OR 1.1; 95% CI 0.4–2.6) [40]. Another case‐control study (538 cases and 800 controls) found no association between hair dye use in the month before or during pregnancy and CBTs (OR 0.96; 95% CI 0.69–1.3) [41].

Data from 1218 cases of CBTs diagnosed between 1976 and 1994 and 2223 matched controls from the general population were included in a multicenter retrospective study from seven countries [42]. Maternal personal use of hair‐coloring agents in the month before or during the pregnancy was not associated with CBTs (OR 1.0; 95% CI 0.83–1.3), nor with astroglial tumor (OR 1.1; 95% CI 0.85–1.4), PNET (OR 1.0; 95% CI 0.71–1.5), and other glial tumors (OR 1.0; 95% CI 0.62–1.0).

The pooled OR for any CBT after periconceptional or pregnancy exposure to hair dye than nonexposed was 1.06 (95% CI 0.88–1.28; *I* ^2^ = 0*%*; chi^2^ *p* value = 0.67) (Figure S2). The certainty of evidence was very low, indicating that whether hair dye exposure before and during pregnancy affects the risk of CBT in the offspring compared with no exposure is very uncertain (Table S3).

#### 3.4.6. Germinal cell tumors

A case‐control study retrospectively examined the relationship between chemical exposure from 6 months before pregnancy to breastfeeding and GCTs in childhood up to 15 years [43]. Maternal exposure to hair dyes 1 month before pregnancy for boys (OR 1.7; 95% CI 1.0–2.8) and during breastfeeding for boys and girls (OR 1.5; 95% CI 1.0–2.2) and for girls alone (OR 1.7; 95% CI 1.1–2.6) was associated with GCTs. The US Servicemen′s Testicular Tumor Environmental and Endocrine Determinants (STEED) study retrospectively examined personal care product use during pregnancy among 527 cases of Testicular germinal cell tumor (TGCT) and 562 controls [44]. Maternal hair dye use ≤ once/week was not associated with TGCT risk (OR 0.80; 95% CI 0.54–1.18). The OR for hair dye use > once/week was not calculated due to small numbers.

A meta‐analysis was not performed due to differences in population sex and investigated GCTs. The certainty of evidence was very low, indicating that whether hair dye exposure before and during pregnancy and breastfeeding affects the risk of GCT in the offspring compared with no exposure is very uncertain (Table S3).

#### 3.4.7. Structural Congenital Heart Defect

The Baltimore–Washington Infant retrospective study investigated factors associated with several major cardiac malformations in Maryland, the District of Columbia, and adjacent counties of northern Virginia between 1981 and 1989 [45]. The study involved 4296 liveborn infants with structural congenital heart defects. For the multiple/multiplex membranous ventricular septal defect, exposure to hair dyes had the largest extra attributable fraction (3.3%) among identified risk factors, indicating the proportion of cases that might be prevented by avoiding the exposure. The certainty of evidence was low, indicating that hair dye exposure during pregnancy may increase the risk of congenital heart defects in the offspring compared with no exposure (Table S3).

#### 3.4.8. Mental Retardation

In a case‐control study on mental retardation and parental occupation [46], parents of 306 mentally retarded children and 322 controls were interviewed about their occupational activities during pregnancy. OR for mental retardation after late pregnancy professional exposure to hair cosmetics and dyes than never exposed was 3.7 (95% CI 0.9–15.1). The certainty of evidence was very low, indicating that whether hair dye exposure during pregnancy affects the risk of mental retardation in the offspring compared with no exposure is very uncertain (Table S3).

## 4. Discussion

### 4.1. Main Findings

Periconceptional or pregnancy exposure to hair dyes was associated with stillbirth, LBW, and lower maternal hormone levels. At least one study observed an association between periconceptional or pregnancy hair dye exposure and adverse outcomes in the offspring, such as a higher risk of allergic disease, leukemia, NB, WT, GCTs, and multiple/multiplex membranous ventricular septal defect; studies that considered the child age at diagnosis observed stronger associations when the analysis was restricted to the first years of life. However, observed associations were supported by low or very low certainty of evidence, being based on single or a few observational studies that preclude causal inference.

### 4.2. Comparison With the Existing Literature: Obstetric Outcomes

The unique study investigating the association between fetal death and hair dye exposure provided concerning data, particularly for hairdressers [27]. Although dose–response analyses showed a growing risk with increased exposure, these observational findings remain hypothesis‐generating and cannot establish a definitive cause‐effect relationship with the association supported by low certainty of evidence. Furthermore, the missing investigation of the hair dye type impedes clarifying the mechanism of action. In this regard, considering that most Japanese beauty salons employ hair dye containing PPD, the authors hypothesized that results likely reflect the exposure to permanent dyes [27]. Aniline derivatives, such as PPD, cause methemoglobinemia following oral ingestion, transdermal absorption, and inhalation [47, 48]. Fetuses are more susceptible to methemoglobinemia due to fetal hemoglobin and lower methemoglobin reductase [49], leading to fetal methemoglobinemia from maternal methemoglobin [50, 51]. On these bases, a transdermal absorption or inhalation of some hair dye compounds could hypothetically induce the placental passage of aniline derivatives, fetal methemoglobinemia, fetal oxygen deprivation, and respiratory or organ failure. However, these mechanisms remain speculative and have not been directly demonstrated in the included studies. Furthermore, the uniquely identified study has limitations that mandate further investigations; approximately half of those who experienced fetal death did not respond to the questionnaire.

Methemoglobinemia may additionally concur in explaining the observed association with LBW [29, 30]. Moreover, ultraviolet filters, phthalate esters, and parabens included in some hair dyes are chemical substances suspected to be endocrine disrupters [52, 53]. Disruptions in prenatal sex steroid hormones may contribute to adverse maternal and fetal outcomes, influencing the secretion of pregestational hormones and increasing the risk of LBW. These endocrine disrupters could hypothetically explain and link the observed association with lower levels of all sex steroid hormones in pregnant women and fetal LBW [31]. Nevertheless, the etiology remains unclear. Moreover, although the meta‐analysis observed the association, caution is mandatory given that only two studies explored LBW risk and preconceptional or pregnancy hair dye exposure with an overall low certainty of evidence [29, 30]. Detailed information on pre‐pregnancy hair dye use, such as frequency and specific product names or types, is missing in one of the two, limiting its value [29].

### 4.3. Comparison With the Existing Literature: Pediatric Outcomes

We identified only two studies investigating and suggesting the relationship between allergic diseases and hair dye exposure [28, 32]. However, this association needs further confirmation. The meta‐analysis for asthma did not confirm an association, and in one study, the diagnosis of allergic diseases was not based on medical records [28]. Moreover, the prevalence of allergic rhinitis is relatively low at 3 years. Finally, the lack of specific information on dye color, method of application, and chemical formulation limits conclusions. Therefore, in line with the GRADE assessment, whether hair dye exposure affects the risk of allergic diseases is very uncertain.

Some chemical compounds in hair dyes are suspected carcinogens [7]. Associations between hair dye exposure and bladder cancer have been reported for the general population [54]. Therefore, the suggested effect of hair dye exposure during the periconceptional period or pregnancy on the risk of some cancers in the offspring is plausible. Notably, the fetus is more vulnerable, particularly during the first trimester [33].

Leukemia is the most common childhood neoplasm [55]. The observed association between ALL and occasional (< 2 times) exposure to hair dyes or hair straightening cosmetics during pregnancy is consistent with the above considerations. Moreover, this association was stronger for exposures during the first trimester [33]. However, the hypothesis of a causal link is limited by the absence of a dose–response pattern based on the entity of exposure, although the effects in the first trimester may not follow a monotonic dose–response pattern [56], as well as the absence of information regarding the type of hair dye [57]. In this regard, uninvestigated differences in the type of hair dyes and the inclusion of cases up to 15 years may explain the unconfirmed association. Therefore, in line with very low certainty of evidence, these findings should be considered hypothesis‐generating and do not allow causal inference. Additionally, the observed association by Rafieemehr et al. [34] was not confirmed by the more recent, although smaller, Iranian case‐control study.

The hypothesis of a potential carcinogenic effect of hair dye on the newborn was further explored by studies investigating NB, WT, and GCT risk. NB is the most common extracranial pediatric solid tumor [58], suggesting a potential role of in utero and neonatal exposures [59]. Consistently, three studies observed a similar increased risk of NB associated with hair dye exposure during pregnancy [35–37], mainly for the first‐/second‐trimester exposure among children under 2 years of age [35] and for permanent hair dye users. The apparent role of age at diagnosis may represent a further hypothesis that warrants investigation for causal inference; a stronger association was observed when the analysis was restricted to diagnosis performed in the first years of life for NB, leukemia, and WT, potentially suggesting a temporal relationship. However, the certainty of evidence was very low, and these findings should therefore be interpreted cautiously.

Finally, chemical disruptors and carcinogens may be part of a more general teratogenic effect that may explain the observed association with multiple/multiplex membranous ventricular septal defect. However, a single study per outcome recommends caution and impedes conclusions.

Overall, although plausible with a possible carcinogenic and teratogenic effect, the observed associations between hair dye exposure during pregnancy and some tumors or congenital defects remain uncertain due to very low or low certainty of evidence.

### 4.4. Strengths and Limitations

Hypothesized carcinogenic, mutagenic, teratogenic, endocrine disruptive, and hypoxic effects are all possible mechanisms explaining the observed associations. Nevertheless, although our findings suggest that personal and professional exposure to hair dye in pregnancy may have a not negligible negative impact on pregnancy and offspring, some limitations recommend caution in supporting a relationship and a direct cause–effect association in line with a low and very low certainty of evidence. A critical limitation of the current evidence is that the assessment of exposure to hair dyes relied exclusively on self‐reported questionnaire surveys or interviews across all included studies. This reliance on subjective reporting introduces a substantial risk of exposure misclassification and recall bias, particularly within the retrospective case‐control studies that dominate the literature. Mothers who have experienced devastating adverse obstetric or pediatric outcomes are inherently more likely to deeply reflect upon and overreport potential chemical exposures during pregnancy compared to healthy controls. Furthermore, the lack of objective exposure tracking prevents an accurate classification of hair dye formulations, such as distinguishing by chemical origin or length of effect, because most studies lacked an in‐depth investigation of the specific products used. This exposure misclassification severely restricts the ability to establish biological plausibility, evaluate dose–response relationships accurately, or define what might constitute a safe hair dye. Moreover, pooled analyses should be interpreted cautiously because of substantial clinical and methodological heterogeneity across studies, including differences in exposure timing, frequency, professional versus personal use, hair dye formulations, and outcome definitions. Therefore, biological plausibility, as well as the definition of potentially safer hair dyes, remains challenging. Finally, some outcomes were investigated by a single study, limiting the generalizability of the findings because of potential sampling bias and precluding a formal assessment of publication bias. Given the limited number of available studies, publication bias was not formally assessed and cannot be excluded. However, the rigorous and systematic approach strengthened the systematic review regardless of the limitations of available evidence. This methodology allowed for identifying all available studies on the association between hair dye exposure in the periconceptional period or pregnancy and adverse obstetric and pediatric outcomes, providing a comprehensive overview of available evidence and related limitations.

## 5. Conclusions

In contrast with most information gathered from the web [4], our results suggest caution in considering the exposure to hair dyes periconceptionally or during pregnancy without risks. The possible associations observed in this review encompass a wide range of adverse outcomes, from fetal death to malignancies in the offspring.

However, these findings are derived primarily from retrospective observational studies and rely on low or very low certainty of evidence. Therefore, further research is required to confirm observed associations and clarify the mechanism of action, defining precisely the type of chemicals involved through more detailed data collection about hair dyes and application methods. Obstetricians, midwives, and hairdressers should know this evidence and its limitations in providing adequate and evidence‐based counseling.

## Author Contributions

Conceptualization: S.U. and N.P. Data curation: S.G., C.C., G.S., and M.B. Formal analysis: S.G., M.B., and P.C.Z. Methodology: S.G. and G.B. Supervision: S.U. and S.G. Writing—original draft: S.U., C.C., and G.S. Writing—review and editing: All authors.

## Funding

No funding was received for this manuscript.

## Disclosure

The authors have nothing to report.

## Conflicts of Interest

The authors declare no conflicts of interest.

## Supporting Information

Additional supporting information can be found online in the Supporting Information section.

## Supporting information


**Supporting Information 1** File S1: Detailed search strategy. Table S1: Characteristics of included studies grouped by investigated outcome. Table S2: Risk of bias assessment. Table S3: GRADE assessment of the certainty of evidence for all outcomes.

**Supporting Information 2** Figure S1: Forest plot of comparison: pregnancy exposure to hair dye versus never exposure; outcome: asthma in the offspring.

**Supporting Information 3** Figure S2: Forest plot of comparison: exposure to hair dye before or during pregnancy versus never exposure; outcome: childhood brain tumors.

**Supporting Information 4**  .

## Data Availability

Data sharing not applicable to this article as no datasets were generated or analyzed during the current study.

## References

[1] Guerra-Tapia A. and Gonzalez-Guerra E. , Hair Cosmetics: Dyes, Actas Dermo-Sifiliográficas. (2014) 105, no. 9, 833–839, 10.1016/j.ad.2014.02.004, 24656996. 24656996

[2] Osterman M. , Hamilton B. , Martin J. A. , Driscoll A. K. , and Valenzuela C. P. , Births: Final Data for 2020, National Vital Statistics Reports. (2021) 70, no. 17, 1–50, 35157571. 35157571

[3] Gera R. , Mokbel R. , Igor I. , and Mokbel K. , Does the Use of Hair Dyes Increase the Risk of Developing Breast Cancer? A Meta-Analysis and Review of the Literature, Anticancer Research. (2018) 38, no. 2, 707–716, 10.21873/anticanres.12276, 29374694. 29374694

[4] NSH.UK , Is It Safe to Use Hair Dye When I’m Pregnant or Breastfeeding?, 2024, https://collingwoodsurgery.nhs.uk/new-york/common-questions/pregnancyis-it-safe-to-use-hair-dye-when-i-am-pregnant-or-breastfeeding.

[5] Henrotin J. , Picot C. , Bouslama M. , Collot-Fertey D. , Radauceanu A. , Labro M. , Larroque B. , Roudot A. C. , Sater N. , Elhkim M. O. , and Lafon D. , Reproductive Disorders in Hairdressers and Cosmetologists: A Meta-Analytical Approach, Journal of Occupational Health. (2015) 57, no. 6, 485–496, 10.1539/joh.15-0068-RA, 26269279. 26269279 PMC6706229

[6] Chye S. M. , Tiong Y. L. , Yip W. K. , Koh R. Y. , Len Y. W. , Seow H. F. , Ng K. Y. , Ranjit D. A. , and Chen S. C. , Apoptosis Induced by Para-Phenylenediamine Involves Formation of ROS and Activation of p38 and JNK in Chang Liver Cells, Environmental Toxicology. (2014) 29, no. 9, 981–990, 10.1002/tox.21828, 23172806. 23172806

[7] IARC Working Group on the Evaluation of Carcinogenic Risks to Humans , Occupational Exposures of Hairdressers and Barbers and Personal Use of Hair Colourants; Some Hair Dyes, Cosmetic Colourants, Industrial Dyestuffs and Aromatic Amines, 1993, International Agency for Research on Cancer. PMC7681637 8207865

[8] Abd-ElZaher M. A. , Fawzy I. A. , Ahmed H. M. , Abd-Allah A. M. , and Gayyed M. F. , Some Toxicological Health Hazards Associated With Subchronic Dermal Exposure to Paraphenylene-Diamine (PPD): An Experimental Study, Egyptian Journal of Forensic Sciences. (2012) 2, no. 3, 105–111, 10.1016/j.ejfs.2012.06.003.

[9] Singh R. L. , Khanna S. K. , Shanker R. , and Singh G. B. , Acute and Short-Term Toxicity Studies on P-Aminodiphenylamine, Veterinary and Human Toxicology. (1986) 28, no. 3, 219–223, 3727352. 3727352

[10] He L. , Michailidou F. , Gahlon H. L. , and Zeng W. , Hair Dye Ingredients and Potential Health Risks From Exposure to Hair Dyeing, Chemical Research in Toxicology. (2022) 35, no. 6, 901–915, 10.1021/acs.chemrestox.1c00427, 35666914. 35666914 PMC9214764

[11] Page M. J. , McKenzie J. E. , Bossuyt P. M. , Boutron I. , Hoffmann T. C. , Mulrow C. D. , Shamseer L. , Tetzlaff J. M. , Akl E. A. , Brennan S. E. , Chou R. , Glanville J. , Grimshaw J. M. , Hróbjartsson A. , Lalu M. M. , Li T. , Loder E. W. , Mayo-Wilson E. , McDonald S. , McGuinness L. , Stewart L. A. , Thomas J. , Tricco A. C. , Welch V. A. , Whiting P. , and Moher D. , The PRISMA 2020 Statement: An Updated Guideline for Reporting Systematic Reviews, British Medical Journal. (2021) 372, 10.1136/bmj.n71, 33782057. PMC8005924 33782057

[12] Moher D. , Liberati A. , Tetzlaff J. , Altman D. G. , and The PRISMA Group , Preferred Reporting Items for Systematic Reviews and Meta-Analyses: The PRISMA Statement, PLOS Medicine. (2009) 6, no. 7, e1000097, 10.1371/journal.pmed.1000097, 19621072. 19621072 PMC2707599

[13] Stroup D. F. , Berlin J. A. , Morton S. C. , Olkin I. , Williamson G. D. , Rennie D. , Moher D. , Becker B. J. , Sipe T. A. , and Thacker S. B. , Meta-Analysis Of Observational Studies In Epidemiology:A Proposal For Reporting, Journal of the American Medical Association. (2000) 283, no. 15, 2008–2012, 10.1001/jama.283.15.2008, 10789670. 10789670

[14] Mintzker Y. , Blum D. , and Adler L. , Replacing PICO in Non-Interventional Studies, BMJ Evidence-Based Medicine. (2023) 28, no. 4, 10.1136/bmjebm-2021-111889, 35017173. 35017173

[15] Morgan R. L. , Whaley P. , Thayer K. A. , and Schünemann H. J. , Identifying the PECO: A Framework for Formulating Good Questions to Explore the Association of Environmental and Other Exposures With Health Outcomes, Environment International. (2018) 121, 1027–1031, 10.1016/j.envint.2018.07.015, 30166065. 30166065 PMC6908441

[16] Jackson J. L. , Kuriyama A. , Anton A. , Choi A. , Fournier J. P. , Geier A. K. , Jacquerioz F. , Kogan D. , Scholcoff C. , and Sun R. , The Accuracy of Google Translate for Abstracting Data From Non–English-Language Trials for Systematic Reviews, Annals of Internal Medicine. (2019) 171, no. 9, 677–679, 10.7326/M19-0891, 31357212. 31357212

[17] Brozek J. L. , Canelo-Aybar C. , Akl E. A. , Bowen J. M. , Bucher J. , Chiu W. A. , Cronin M. , Djulbegovic B. , Falavigna M. , Guyatt G. H. , Gordon A. A. , Hilton Boon M. , Hutubessy R. C. W. , Joore M. A. , Katikireddi V. , LaKind J. , Langendam M. , Manja V. , Magnuson K. , Mathioudakis A. G. , Meerpohl J. , Mertz D. , Mezencev R. , Morgan R. , Morgano G. P. , Mustafa R. , O′Flaherty M. , Patlewicz G. , Riva J. J. , Posso M. , Rooney A. , Schlosser P. M. , Schwartz L. , Shemilt I. , Tarride J. E. , Thayer K. A. , Tsaioun K. , Vale L. , Wambaugh J. , Wignall J. , Williams A. , Xie F. , Zhang Y. , Schünemann H. J. , and GRADE Working Group , GRADE Guidelines 30: The GRADE Approach to Assessing the Certainty of Modeled Evidence—An Overview in the Context of Health Decision-Making, Journal of Clinical Epidemiology. (2021) 129, 138–150, 10.1016/j.jclinepi.2020.09.018, 32980429. 32980429 PMC8514123

[18] van Genderen M. E. , Carels G. , Lonnee E. R. , and Dees A. , Severe Facial Swelling in a Pregnant Woman After Using Hair Dye, BMJ Case Reports. (2014) 2014, bcr2013202562, 10.1136/bcr-2013-202562, 24686800. PMC3975514 24686800

[19] Ferreira J. D. , Couto A. C. , Alves L. C. , Oliveira M. , and Koifman S. , Exposições Ambientais E Leucemias Na Infância No Brasil: Uma Análise Exploratória De Sua Associação, Revista Brasileira de Estudos de População. (2012) 29, no. 2, 477–492, 10.1590/S0102-30982012000200014.

[20] Su Y. H. , Cheng Q. J. , Huang Z. X. , Zhao B. , Chen J. H. , Yu X. S. , Peng X.-Q. , Zhao B. , Shi M.-M. , Chen J. , Liu H.-F. , Ke X.-Y. , and Zhao B. H. , Factors Associated With Internal Phthalate Exposure Among Pregnant Women, DEStech Transactions on Environment, Energy and Earth Sciences, 2017, DEStech Publications, 10.12783/dteees/sses/icfse2016/10616.

[21] Kulahci P. , Kartal B. , and Bulbul T. , Exposure of Pregnant Women to Chemicals and Cosmetic Products, Journal of Education and Research in Nursing.(2022) 19, no. 4, 403–408, 10.5152/jern.2022.99896.

[22] Chua-Gocheco A. , Bozzo P. , and Einarson A. , Safety of Hair Products During Pregnancy: Personal Use and Occupational Exposure, Canadian Family Physician. (2008) 54, no. 10, 1386–1388, 18854462. 18854462 PMC2567273

[23] Chabert M. C. , Perrin J. , Berbis J. , Bretelle F. , Adnot S. , and Courbiere B. , Lack of Information Received by a French Female Cohort Regarding Prevention Against Exposure to Reprotoxic Agents During Pregnancy, European Journal of Obstetrics & Gynecology and Reproductive Biology. (2016) 205, 15–20, 10.1016/j.ejogrb.2016.07.504, 27552174. 27552174

[24] Shawahna R. , Saleh R. , Owiwi L. , Abdi A. , Bani-Odeh D. , Maqboul I. , Hijaz H. , and Jaber M. , Breastmilk Cadmium Levels and Estimated Infant Exposure: A Multicenter Study of Associated Factors in a Resource-Limited Country, International Breastfeeding Journal. (2023) 18, no. 1, 10.1186/s13006-023-00574-0, 37501132. PMC10375743 37501132

[25] Ziadat A. H. , Disabilities of Children in Correlation to the Usage of Hair Dye Among Pregnant Women, International Journal of Pharmacology. (2010) 6, no. 4, 487–493, 10.3923/ijp.2010.487.493.

[26] Fan C. L. , Luo J. Y. , Gong W. J. , Liu X. Q. , Zhou S. J. , Zhang F. F. , Zeng J. , Li H. , and Feng N. , Nested Case-Control Study on Associated Factors for Anemia During Pregnancy, Chinese Journal of Epidemiology. (2017) 38, no. 9, 1269–1273, 10.3760/cma.j.issn.0254-6450.2017.09.025. 28910945

[27] Ooka T. , Horiuchi S. , Shinohara R. , Kojima R. , Akiyama Y. , Miyake K. , Otawa S. , Yokomichi H. , Yamagata Z. , and on behalf of the Japan Environment and Children’s Study Group , Association Between Maternal Exposure to Chemicals During Pregnancy and the Risk of Foetal Death: The Japan Environment and Children′s Study, International Journal of Environmental Research and Public Health. (2021) 18, no. 22, 11748, 10.3390/ijerph182211748, 34831503. 34831503 PMC8618242

[28] Kojima R. , Shinohara R. , Horiuchi S. , Otawa S. , Yokomichi H. , Akiyama Y. , Ooka T. , Miyake K. , Yamagata Z. , and Japan Environment and Children’s Study Group , Association Between Gestational Hair Dye Use and Allergies at 3 Years Old: The Japan Environment and Children′s Study, Environmental Research. (2021) 201, 111530, 10.1016/j.envres.2021.111530, 34171376. 34171376

[29] Jiang C. , Hou Q. , Huang Y. , Ye J. , Qin X. , Zhang Y. , Meng W. , Wang Q. , Jiang Y. , Zhang H. , Li M. , Mo Z. , and Yang X. , The Effect of Pre-Pregnancy Hair Dye Exposure on Infant Birth Weight: A Nested Case-Control Study, BMC Pregnancy Childbirth. (2018) 18, no. 1, 10.1186/s12884-018-1782-5, 29743046. PMC5944114 29743046

[30] Kazemi Shishavan M. , Sayyah-Melli M. , Rashidi M. R. , Mostafa Gharabaghi P. , Ghojazadeh M. , Rahmani V. , and Tahmasebi Z. , The Association of Hair Coloring During Pregnancy With Pregnancy and Neonatal Outcomes: A Cross-Sectional Study, International Journal of Women′s Health and Reproduction Sciences. (2021) 9, no. 2, 130–135, 10.15296/ijwhr.2021.23.

[31] Rivera-Núñez Z. , Ashrap P. , Barrett E. S. , Llanos A. A. M. , Watkins D. J. , Cathey A. L. , Vélez-Vega C. M. , Rosario Z. , Cordero J. F. , Alshawabkeh A. , and Meeker J. D. , Personal Care Products: Demographic Characteristics and Maternal Hormones in Pregnant Women From Puerto Rico, Environmental Research. (2022) 206, 112376, 10.1016/j.envres.2021.112376, 34798118. 34798118 PMC8810700

[32] Tokinobu A. , Tanaka K. , Arakawa M. , and Miyake Y. , Pre- and Postnatal Maternal Hair Dye Use and Risk of Wheeze and Asthma in 5-Year-Old Japanese Children: The Kyushu Okinawa Maternal and Child Health Study, International Journal of Environmental Health Research. (2023) 33, no. 12, 1697–1705, 10.1080/09603123.2022.2120189, 36062394. 36062394

[33] Couto A. C. , Ferreira J. D. , Rosa A. C. S. , Pombo-de-Oliveira M. S. , Koifman S. , and Brazilian Collaborative Study Group of Infant Acute Leukemia , Pregnancy, Maternal Exposure to Hair Dyes and Hair Straightening Cosmetics, and Early Age Leukemia, Chemico-Biological Interactions. (2013) 205, no. 1, 46–52, 10.1016/j.cbi.2013.05.012, 23747844. 23747844

[34] Rafieemehr H. , Calhor F. , Esfahani H. , and Ghorbani G. S. , Risk of Acute Lymphoblastic Leukemia: Results of a Case-Control Study, Asian Pacific Journal of Cancer Prevention.(2019) 20, no. 8, 2477–2483, 10.31557/APJCP.2019.20.8.2477, 31450923. 31450923 PMC6852832

[35] Parodi S. , Merlo D. F. , Ranucci A. , Miligi L. , Benvenuti A. , Rondelli R. , Magnani C. , Haupt R. , and SETIL Working Group , Risk of Neuroblastoma, Maternal Characteristics and Perinatal Exposures: The SETIL Study, Cancer Epidemiology. (2014) 38, no. 6, 686–694, 10.1016/j.canep.2014.09.007, 25280392. 25280392

[36] McCall E. E. , Olshan A. F. , and Daniels J. L. , Maternal Hair Dye Use and Risk of Neuroblastoma in Offspring, Cancer Causes & Control. (2005) 16, no. 6, 743–748, 10.1007/s10552-005-1229-y, 16049813. 16049813

[37] Kramer S. , Ward E. , Meadows A. T. , and Malone K. E. , Medical and Drug Risk Factors Associated With Neuroblastoma: A Case-Control Study, Journal of the National Cancer Institute. (1987) 78, no. 5, 797–804, 10.1093/jnci/78.5.797. 3471992

[38] Bunin G. R. , Kramer S. , Marrero O. , and Meadows A. T. , Gestational Risk Factors for Wilms′ Tumor: Results of a Case-Control Study, Cancer Research. (1987) 47, no. 11, 2972–2977, 3032418. 3032418

[39] Olshan A. F. , Breslow N. E. , Falletta J. M. , Grufferman S. , Pendergrass T. , Robison L. L. , Waskerwitz M. , Woods W. G. , Vietti T. J. , and Hammond G. D. , Risk Factors for Wilms Tumor: Report From the National Wilms Tumor Study, Cancer. (1993) 72, no. 3, 938–944, 10.1002/1097-0142(19930801)72:3<938::AID-CNCR2820720345>3.0.CO;2-C. 8392906

[40] Bunin G. R. , Buckley J. D. , Boesel C. P. , Rorke L. B. , and Meadows A. T. , Risk Factors for Astrocytic Glioma and Primitive Neuroectodermal Tumor of the Brain in Young Children: A Report From the Children′s Cancer Group, Cancer Epidemiology, Biomarkers & Prevention. (1994) 3, no. 3, 197–204. 8019366

[41] Holly E. A. , Bracci P. M. , Hong M. , Mueller B. A. , and Preston-Martin S. , West Coast Study Of Childhood Brain Tumours And Maternal Use Of Hair-Colouring Products, Paediatric and Perinatal Epidemiology. (2002) 16, no. 3, 226–235, 10.1046/j.1365-3016.2002.00420.x, 12123435. 12123435

[42] Efird J. T. , Holly E. A. , Cordier S. , Mueller B. A. , Lubin F. , Filippini G. , Peris-Bonet R. , McCredie M. , Arslan A. , Bracci P. , and Preston-Martin S. , Beauty product-related exposures and childhood brain tumors in seven countries: results from the SEARCH International Brain Tumor Study, Journal of Neuro-Oncology. (2005) 72, no. 2, 133–147, 10.1007/s11060-004-3121-0, 15925993. 15925993

[43] Chen Z. , Robison L. , Giller R. , Krailo M. , Davis M. , Davies S. , and Shu X. O. , Environmental Exposure To Residential Pesticides, Chemicals, Dusts, Fumes, And Metals, And Risk Of Childhood Germ Cell Tumors, International Journal of Hygiene and Environmental Health. (2006) 209, no. 1, 31–40, 10.1016/j.ijheh.2005.08.001, 16373200. 16373200

[44] Ghazarian A. A. , Trabert B. , Robien K. , Graubard B. I. , and McGlynn K. A. , Maternal Use of Personal Care Products During Pregnancy and Risk of Testicular Germ Cell Tumors in Sons, Environmental Research. (2018) 164, 109–113, 10.1016/j.envres.2018.02.017, 29482183. 29482183 PMC5911208

[45] Wilson P. D. , Loffredo C. A. , Correa-Villasenor A. , and Ferencz C. , Attributable Fraction for Cardiac Malformations, American Journal of Epidemiology. (1998) 148, no. 5, 414–423, 10.1093/oxfordjournals.aje.a009666. 9737553

[46] Roeleveld N. , Zielhuis G. A. , and Gabreels F. , Mental Retardation and Parental Occupation: A Study on the Applicability of Job Exposure Matrices, Occupational & Environmental Medicine. (1993) 50, no. 10, 945–954, 10.1136/oem.50.10.945, 8217856. PMC1035526 8217856

[47] Kearney T. E. , Manoguerra A. S. , and Dunford J. V. , Chemically Induced Methemoglobinemia From Aniline Poisoning, Western Journal of Medicine. (1984) 140, no. 2, 282–286, 6730477. 6730477 PMC1021626

[48] de Vere F. , Moores R. , Dhadwal K. , and Karra E. , A Severe Case of Methaemoglobinaemia in a Brazilian Hairdresser, BMJ Case Reports. (2020) 13, no. 1, 10.1136/bcr-2019-232735, 31969408, e232735. PMC7021157 31969408

[49] Herzog P. and Feig S. A. , Methaemoglobinaemia in the Newborn Infant, Clinical Haematology. (1978) 7, no. 1, 75–83, 10.1016/S0308-2261(21)00571-3. 657603

[50] Mohorovic L. , The Role of Methemoglobinemia in Early and Late Complicated Pregnancy, Medical Hypotheses. (2007) 68, no. 5, 1114–1119, 10.1016/j.mehy.2006.09.053, 17112681. 17112681

[51] Mohorovic L. , Petrovic O. , Haller H. , and Micovic V. , Pregnancy Loss and Maternal Methemoglobin Levels: An Indirect Explanation of the Association of Environmental Toxics and Their Adverse Effects on the Mother and the Fetus, International Journal of Environmental Research and Public Health. (2010) 7, no. 12, 4203–4212, 10.3390/ijerph7124203, 21318003. 21318003 PMC3037049

[52] Dodson R. E. , Nishioka M. , Standley L. J. , Perovich L. J. , Brody J. G. , and Rudel R. A. , Endocrine Disruptors and Asthma-Associated Chemicals in Consumer Products, Environmental Health Perspectives. (2012) 120, no. 7, 935–943, 10.1289/ehp.1104052, 22398195. 22398195 PMC3404651

[53] Witorsch R. J. and Thomas J. A. , Personal Care Products and Endocrine Disruption: A Critical Review of the Literature, Critical Reviews in Toxicology. (2010) 40, 1–30, 10.3109/10408444.2010.515563, 20932229. 20932229

[54] Kim K. H. , Kabir E. , and Jahan S. A. , The Use of Personal Hair Dye and Its Implications for Human Health, Environment International. (2016) 89-90, 222–227, 10.1016/j.envint.2016.01.018, 26895479. 26895479

[55] International Agency for Research on Cancer , Estimated Age-Standardized Incidence Rates (World) in 2020, World, Both Sexes, Ages 0-19, 2024, https://gco.iarc.who.int/today/en/dataviz/bars?types=0_1%26mode=population.

[56] Heindel J. J. , Animal Models for Probing the Developmental Basis of Disease and Dysfunction Paradigm, Basic & Clinical Pharmacology & Toxicology. (2008) 102, no. 2, 76–81, 10.1111/j.1742-7843.2007.00184.x, 18226058. 18226058

[57] Mazzei J. L. , da Silva D. N. , Oliveira V. , Hosomi R. Z. , do Val R. R. , Pestana C. B. , and Felzenszwalb I. , Absence of Mutagenicity of Acid Pyrogallol-Containing Hair Gels, Food and Chemical Toxicology. (2007) 45, no. 4, 643–648, 10.1016/j.fct.2006.10.013, 17140719. 17140719

[58] Maris J. M. , Recent Advances in Neuroblastoma, New England Journal of Medicine. (2010) 362, no. 23, 2202–2211, 10.1056/NEJMra0804577, 20558371. 20558371 PMC3306838

[59] London W. B. , Castleberry R. P. , Matthay K. K. , Look A. T. , Seeger R. C. , Shimada H. , Thorner P. , Brodeur G. , Maris J. M. , Reynolds C. P. , and Cohn S. L. , Evidence for an Age Cutoff Greater Than 365 Days for Neuroblastoma Risk Group Stratification in the Children′s Oncology Group, Journal of Clinical Oncology. (2005) 23, no. 27, 6459–6465, 10.1200/JCO.2005.05.571, 16116153. 16116153

