# Supplementary material for: Hair Dyes in Pregnancy: A Systematic Review and Meta‐Analysis
Source: J Pregnancy. 2026 Sep 1;2026:9361797. doi: 10.1155/jp/9361797 (PMC13535154; doi:10.1155/jp/9361797)
Supplement: Supplementary file 1 [file JP-2026-9361797-s001.docx]

**Supplementary material S1**

***Detailed search strategy***

Search Query used in all databases:

**(periconceptional OR conceptional OR conception OR conceptions OR fertilization OR fertilizations OR prenatal OR postnatal OR pregnancy OR pregnancies OR gestation OR gestational OR pregnant OR lactation OR lactating OR newborn OR neonatal) AND ("Hair Dyes" OR "Hair Dye" OR "Hair Coloring Agents" OR "Hair Coloring Agent" OR "Hair Colorants" OR "Hair Colorant" OR ((henna OR Lawsonia) AND hair))**

*The search strategy was performed without MeSH terms to include non-indexed more recent records and to make the search query replicable in all electronic databases.*

**Table S1**. Characteristics of included studies grouped by investigated outcome.

| **Table S1. Characteristics of included studies grouped by investigated outcome.** | | | | | |
| --- | --- | --- | --- | --- | --- |
| **FETAL DEATH** | | | | | |
| **Author, year** | **Study design** | **Population** | **Nation** | **Results** | **Possible source of bias** |
| Ooka, 2021 | Retrospective cohort study | 97,415 (101,446 fetuses) | Japan | - Association with stillbirth: 1-6 times/week use vs. never (aOR 4.92, CI 1.53-15.78); everyday use vs. never (aOR 7.59, CI 2.32-24.85) - Escalation of the risk with frequency of exposure (p for trend: 0.0011) - Association between hairdressing and stillbirths (aOR: 3.56, CI: 1.54–8.24) - Personal “quite often” use OR for home use was 1.98 (CI: 0.62–6.29); OR for use in hair salons: 1.92 (CI: 0.88–4.22)) | - Exposure based on questionnaire surveys - Half of the women who experienced spontaneous abortions or stillbirths did not respond - Not collected the specific type of used hair dye |
| **LOW BIRTH WEIGHT AND NEONATAL COMPLICATIONS** | | | | | |
| **Author, year** | **Study design** | **Population** | **Nation** | **Results** | **Possible source of bias** |
| Jiang, 2018 | Case-control study | 4,978 | China | Association of pre-pregnancy hair dye exposure with Low birth weight (OR 1.71, CI 1.01-2.92) | - Exposure based on questionnaire surveys - Not collected the specific type of used hair dye |
| Kazemi Shishavan, 2020 | Cross-sectional study | 2,040 | Tabriz | Associations with higher risk of Low birth weight (P<0.001) for periconceptional or third trimester hair coloring than never exposure or exposure in the first or second trimester. | - Exposure based on questionnaire surveys - Exclusion of professional hairdressers and beauty salon workers |
| **HORMONE LEVEL IN THE MOTHER** | | | | | |
| **Author, year** | **Study design** | **Population** | **Nation** | **Results** | **Possible source of bias** |
| Rivera-N [úñez](https://pubmed.ncbi.nlm.nih.gov/?term=Rivera-N%C3%BA%C3%B1ez+Z&cauthor_id=34798118), 2022 | Prospective cohort study | 1,070 | Puerto Rico | Lower levels of all sex steroid hormones in pregnancy: SHBG (%Δ = −7.1, CI −12.4, -1.8), E3 (%Δ = −23.2, CI −32.2,-13.0), progesterone (%Δ = −21.5, CI −29.4,-12.9) and testosterone (%Δ = −21.5, CI: −33.1,-7.8) | - Exposure based on questionnaire surveys - Data on length of exposure not investigated |
| **ALLERGIC DISEASE** | | | | | |
| **Author, year** | **Study design** | **Population** | **Nation** | **Results** | **Possible source of bias** |
| Kojima, 2021 | Retrospective cohort study | 77,303 | Japan | Association between “quite often” home hair dye use and allergic rhinitis in children (OR 1.78, 95% CI 1.22–2.60). | - Exposure based on questionnaire surveys |
| Tokinobu, 2023 | Retrospective cohort study | 1,199 | Japan | Hair dye use during pregnancy is associated with an increased risk of neonatal wheeze (aOR 1.44, CI 1.02-2.02) and asthma (aOR 1.51, CI 1.00-2.25). | - Exposure based on questionnaire surveys - Non representative population - Not collected the specific type of used hair dye |
| **LEUKEMIA** | | | | | |
| **Author, year** | **Study design** | **Population** | **Nation** | **Results** | **Possible source of bias** |
| Couto, 2013 | Case-control study | 176 Acute lymphatic leukemia  55 Acute myeloid leukemia  419 controls | Brazil | Association between hair dye use in the first trimester and Acute lymphatic leukemia (aOR 1.78, CI 1.13-2.81)  Association between hair dye use during breastfeeding and acute myeloid leukemia (aOR 2.43, CI: 1.13–5.22) | - Exposure based on questionnaire surveys - Hair dyes exposure was merged with hair straightening cosmetics exposure - Not collected the specific type of used hair dye |
| [Rafieemehr](https://pubmed.ncbi.nlm.nih.gov/?term=Rafieemehr%20H%5BAuthor%5D), 2019 | Case-control study | 125 Acute lymphatic leukemia  130 controls | Iran | No association with Acute lymphatic leukemia (OR 0.87, CI 0.32 - 2.37) | - Exposure based on questionnaire surveys - Small sample size - Diagnosis up to 15 years of age |
| **NEUROBLASTOMA** | | | | | |
| **Author, year** | **Study design** | **Population** | **Nation** | **Results** | **Possible source of bias** |
| Parodi, 2014 | Case-control study | 153 cases  1044 controls | Italy | Permanent hair dyes use in the first and second trimester was associated with Neuroblastoma, especially for diagnosis in the 0–17-month infants (OR = 5.5, CI: 1.0–29.3) | - Exposure based on questionnaire surveys |
| McCall, 2005 | Case-control study | 538 cases  504 controls | United States | - Use of any hair dye in the month before or during pregnancy is associated with Neuroblastoma (OR 1.6, CI 1.2-2.2) - Temporary hair dye use had a stronger association (OR 2.0, CI 1.1-3.7) than permanent dye | - Exposure based on questionnaire surveys |
| Kramer, 1987 | Case-control study | 104 cases  104 controls | United States | Use of any hair dye during pregnancy was associated with Neuroblastoma (OR 3.0, CI: 1.64-5.48) | - Exposure based on questionnaire surveys - Not collected the specific type of used hair dye |
| **WILMS TUMOR** | | | | | |
| **Author, year** | **Study design** | **Population** | **Nation** | **Results** | **Possible source of bias** |
| Bunin, 1987 | Case-control study | 88 matched case–control pairs | United States | Association between use of hair dyes in the year before childbirth and Wilms tumor (OR 3.6, CI 1.4–10.2) | - Exposure based on questionnaire surveys |
| Olshan, 1993 | Case-control study | 200 cases  233 controls | Canada | No significant association with Wilms tumor (OR 1.37, CI 0.66-2.85) | - Exposure based on questionnaire surveys |
| **CHILDHOOD BRAIN TUMORS** | | | | | |
| **Author, year** | **Study design** | **Population** | **Nation** | **Results** | **Possible source of bias** |
| Bunin, 1994 | Case-control study | 321 matched case–control pairs | United States | No association with astrocytic glioma (OR 0.7, CI 0.3–1.6) or primitive neuroectodermal tumor of the brain (OR 1.1, CI 0.4–2.6) | - Exposure based on questionnaire surveys |
| Holly, 2002 | Case-control study | 538 cases  800 controls | United States | No association with childhood brain tumors (OR 0.96, CI 0.69–1.3) | - Exposure based on questionnaire surveys - Not collected the specific type of used hair dye |
| Efird, 2005 | Case-control study | 1218 cases  2223 controls | 7 countries | No association with childhood brain tumors (OR 1.0, CI 0.83–1.3) or with astroglia (OR 1.1, CI 0.85–1.4), Primitive NeuroEctodermal Tumor of the brain (OR 1.0, CI: 0.71–1.5) and other glial subtypes (OR 1.0, CI 0.62–1.0) | - Exposure based on questionnaire surveys |
| **GERMINAL CELL TUMOR** | | | | | |
| **Author, year** | **Study design** | **Population** | **Nation** | **Results** | **Possible source of bias** |
| Chen, 2006 | Case-control study | 272 cases  418 controls | New Zealand | Association between hair dyes use in pregnancy and Germinal cell tumor only for boys (OR 1.7, CI 1.0–2.8)  Use during breastfeeding associated with Germinal cell tumor for boys and girls (OR 1.5, CI 1.0–2.2) | - Exposure based on questionnaire surveys - Low response rate among controls - Possible exclusion of lower socioeconomic status families from phone interview |
| Ghazarian, 2018 | Case-control study | 527 cases  562 controls | United States | No association with Testicular Germinal cell tumor (OR 0.80, CI 0.54–1.18) | - Exposure based on questionnaire surveys - Not collected the specific type of used hair dye |
| **STRUCTURAL CONGENITAL HEART DEFECT** | | | | | |
| **Author, year** | **Study design** | **Population** | **Nation** | **Results** | **Possible source of bias** |
| Wilson, 1998 | Case-control study | 4,296 cases  3,572 controls | United States | Extra attributable fraction of hair dyes for Multiple/multiplex membranous ventricular septal defect of 3.3% | - Exposure based on questionnaire surveys |
| **MENTAL RETARDATION** | | | | | |
| **Author, year** | **Study design** | **Population** |  | **Results** | **Possible source of bias** |
| Roeleveld, 1993 | Case-control study | 306 cases  322 controls | Danmark | Association with mental retardation in children (OR = 3.7, CI 0.9-15.1) | - Exposure based on questionnaire surveys |
| OR, Odds Ratio; aOR, adjusted Odds Ratio | | | | | |

**Table S2**. Risk of bias assessment.

| **Table S2. Risk of bias assessment.** | | | |
| --- | --- | --- | --- |
|  | **Newcastle-Ottawa Scale Score** | | |
| **Authors (year)** | **Selection** | **Comparability** | **Outcome** |
| Kojima et al. 2021 | *** | * | ** |
| Ooka et al. 2021 | *** | * | ** |
| Jiang et al.2018 | **** | * | *** |
| Couto et al. 2013 | **** | * | *** |
| [Rafieemehr](https://pubmed.ncbi.nlm.nih.gov/?term=Rafieemehr%20H%5BAuthor%5D) et al. 2019 | *** | * | *** |
| Parodi et al. 2014 | *** | * | *** |
| McCall et al. 2005 | *** | * | *** |
| Bunin et al. 1987 | **** | * | *** |
| Olshan et al. 1993 | *** | * | *** |
| Bunin et al. 1994 | *** | * | *** |
| Holly et al. 2002 | **** | * | *** |
| Efird et al. 2005 | **** | * | *** |
| Chen et al. 2006 | **** | * | *** |
| Ghazarian et al. 2018 | **** | * | *** |
| Wilson et al. 1998 | **** | * | *** |
| Roeleveld et al. 1993 | *** | * | *** |
| Rivera-N[úñez](https://pubmed.ncbi.nlm.nih.gov/?term=Rivera-N%C3%BA%C3%B1ez+Z&cauthor_id=34798118) et al. 2022 | *** | * | *** |
| Kazemi Shishavan et al. 2020 | **** | * | *** |
| Kramer et al. 1987 | **** | * | *** |
| Tokinobu et al. 2023 | *** | * | ** |

**Table S3**. GRADE assessment of the certainty of evidence for all outcomes.

| **Table S3. GRADE assessment of the certainty of evidence for all outcomes.** | | | | | | | | |
| --- | --- | --- | --- | --- | --- | --- | --- | --- |
| **Outcomes** | Participants (studies) | Risk of bias | Inconsistency | Indirectness | Imprecision | Publication bias | Overall certainty of evidence | Statement |
| **Fetal death** | 97.415 (1 non-randomised study) | serious | not valuable | not serious | not serious | dose response gradient | ⨁⨁◯◯  Low | Hair dye exposure during the first and second/third trimester of pregnancy may increase the risk of fetal death compared with no exposure. |
| **Low birth weight** | 7.018 (2 non-randomised studies) | not serious | not serious | not serious | not serious | none | ⨁⨁◯◯  Low | Hair dye exposure during preconception may increase the risk of LBW compared with no exposure. |
| **Maternal hormones** | 1.070 (1 non-randomised study) | serious | not valuable | not serious | serious | none | ⨁◯◯◯ Very low | Whether hair dye exposure during pregnancy reduces hormone levels compared with no exposure is very uncertain. |
| **Allergic diseases** | 78.502 (2 non-randomised studies) | serious | serious | not serious | serious | none | ⨁◯◯◯ Very low | Whether hair dye exposure during pregnancy affects the risk of asthma in the offspring compared with no exposure is very uncertain. |
| **Leukemia** | 905 (2 non-randomised studies) | serious | serious | not serious | serious | none | ⨁◯◯◯ Very low | Whether hair dye exposure during pregnancy affects the risk of leukemia in the offspring compared with no exposure is very uncertain. |
| **Neuroblastoma** **- Any hair dye** | 2.447 (3 non-randomised studies) | serious | not serious | not serious | not serious | none | ⨁◯◯◯ Very low | Whether hair dye exposure before and during pregnancy increases the risk of NB in the offspring compared with no exposure is very uncertain. |
| **Wilms tumor** | 609 (2 non-randomised studies) | serious | serious | not serious | serious | none | ⨁◯◯◯ Very low | Whether hair dye exposure before or during pregnancy increases the risk of WT in the offspring compared with no exposure is very uncertain. |
| **Childhood brain tumors** | 5.421 (3 non-randomised studies) | serious | not serious | not serious | not serious | none | ⨁◯◯◯ Very low | Whether hair dye exposure before and during pregnancy affects the risk of CBT in the offspring compared with no exposure is very uncertain. |
| **Germinal cell tumor** | 1.779 (2 non-randomised studies) | not serious | serious | not serious | serious | none | ⨁◯◯◯ Very low | Whether hair dye exposure before and during pregnancy and breastfeeding affects the risk of GCT in the offspring compared with no exposure is very uncertain. |
| **Structural congenital heart defect** | 7.868 (1 non-randomised study) | not serious | not valuable | not serious | not serious | none | ⨁⨁◯◯ Low | Hair dye exposure during pregnancy may increase the risk of congenital heart defects in the offspring compared with no exposure. |
| **Mental retardation** | 628 (1 non-randomised study) | serious | not valuable | not serious | serious | none | ⨁◯◯◯ Very low | Whether hair dye exposure during pregnancy affects the risk of mental retardation in the offspring compared with no exposure is very uncertain. |
